# Differential Modulation of TNF-α–Induced Apoptosis by *Neisseria meningitidis*

**DOI:** 10.1371/journal.ppat.1000405

**Published:** 2009-05-01

**Authors:** Ala-Eddine Deghmane, Carole Veckerlé, Dario Giorgini, Eva Hong, Corinne Ruckly, Muhamed-Kheir Taha

**Affiliations:** Institut Pasteur, Invasive Bacterial Infections Unit, Paris, France; Northwestern University Feinberg School of Medicine, United States of America

## Abstract

Infections by *Neisseria meningitidis* show duality between frequent asymptomatic carriage and occasional life-threatening disease. Bacterial and host factors involved in this balance are not fully understood. Cytopathic effects and cell damage may prelude to pathogenesis of isolates belonging to hyper-invasive lineages. We aimed to analyze cell–bacteria interactions using both pathogenic and carriage meningococcal isolates. Several pathogenic isolates of the ST-11 clonal complex and carriage isolates were used to infect human epithelial cells. Cytopathic effect was determined and apoptosis was scored using several methods (FITC-Annexin V staining followed by FACS analysis, caspase assays and DNA fragmentation). Only pathogenic isolates were able to induce apoptosis in human epithelial cells, mainly by lipooligosaccharide (endotoxin). Bioactive TNF-α is only detected when cells were infected by pathogenic isolates. At the opposite, carriage isolates seem to provoke shedding of the TNF-α receptor I (TNF-RI) from the surface that protect cells from apoptosis by chelating TNF-α. Ability to induce apoptosis and inflammation may represent major traits in the pathogenesis of *N. meningitidis*. However, our data strongly suggest that carriage isolates of meningococci reduce inflammatory response and apoptosis induction, resulting in the protection of their ecological niche at the human nasopharynx.

## Author Summary

Acquisition of *Neisseria meningitidis* often leads to asymptomatic colonization (carriage) and rarely results in invasive disease associated with tissue injury. The reasons that make disease-associated isolates (pathogenic isolates) but not asymptomatic carriage isolates able to invade the host to establish disease are not understood. Isolates belonging to the ST-11 clonal complex are most frequently associated with the disease and rarely found in carriers. These hyper-invasive isolates may be able to induce cytopathic effects in target cells. We aimed to investigate the cytopathic effect of meningococcal isolates on epithelial cells using both ST-11 pathogenic isolates and carriage isolates. We showed that cytopathic effects were strongly associated with pathogenic isolates and infected cells exhibited features of apoptosis. This effect is mainly mediated by bacterial endotoxin (lipooligosaccharide) and involved an autocrine signaling mechanism of secreted TNF-α through its receptor TNF-RI. In contrast, carriage isolates down-regulate TNF-RI on the surface of infected cells by increasing TNF-RI shedding into the medium. We suggest that chelating secreted TNF-α protects cells from apoptosis. Our results unravel a differential modulation of TNF-α signaling by meningococcal isolates leading to cell survival or death and would therefore contribute to better understanding of the duality between carriage and invasiveness.

## Introduction

*Neisseria meningitidis* is the causative agent of meningococcal meningitis and fulminant sepsis. It is a common inhabitant of the human nasopharynx, being asymptomatically carried by approximately 10% of the population, worldwide. The incidence of meningococcal disease varies from 1 to 50 cases per 100,000. The reported fatality rate in meningococcal meningitis is about 10% and up to 20% of survivors suffer from sequelae [1]. While in industrialized countries, meningococcal disease occurs usually as sporadic cases, large epidemics occur periodically in the “meningitis belt” of sub-Saharan Africa (from Senegal to Ethiopia). There is increasing evidence that invasive meningococcal infections

lead to cytopathic effects that often result in marked tissue and cell damage mainly characterized by the breakdown of cell tight junctions, the deterioration of the cell layers, changes in normal cell morphology, and loss of cells [2]–[8]. These observations are consistent with the extensive tissue damage and cell death seen in autopsy material from fatal human cases [9]. Tissue damage may occur through apoptosis and/or necrosis. Various components of the bacterial cell wall are capable of activating proinflammatory response, notably the meningococcal lipooligosaccharide (LOS), the major structural component of the outer membrane.

Apoptosis plays an important role in the pathogenesis of several infectious agents that induce or block this process [10]–[14]. Pathogenic *Neisseriae* have been shown to induce the expression of apoptosis-related genes and to trigger apoptosis in different cell types [15]–[19]. However, the mechanisms leading to this programmed cell death are still poorly understood. Interestingly, conflicting evidences exist on the ability of the neisserial porin PorB to induce or to protect cells from apoptosis [16],[17],[20].

Moreover, meningococci-cell interaction is complicated by the high variability of meningococcal isolates. Multilocus sequence typing (MLST) analysis classifies meningococcal isolates according to polymorphisms in seven housekeeping genes. Meningococcal isolates can be clustered into clonal complexes comprising closely related isolates varying by no more than two loci [21]. Molecular epidemiology studies comparing the overall diversity between the pathogenic and carriage population, show that isolates from asymptomatic carriage are more diverse than those from invasive disease [22],[23]. Despite the diversity of carried meningococci, only a limited number of clonal complexes, termed the hyper-invasive lineages, are responsible for most reported disease [22]–[24]. Among these clonal complexes, isolates belonging to the clonal complex ST-11 seem to be significantly associated with the disease and rarely found in carriers [22]. Moreover, we have recently shown that isolates of the ST-11 clonal complex positively correlated with fatal outcome, a higher virulence for mice and a higher damage to human epithelial cells, disregarding their serogroups [25].

The aim of this work was to explore the apoptosis pathway induced by hyper-invasive ST-11 isolates of *N. meningitidis* in comparison to non invasive carriage isolates.

## Results

### Prevalence of cytopathogenicity among ST-11 pathogenic and carriage isolates of Nm

**Figure 1:**
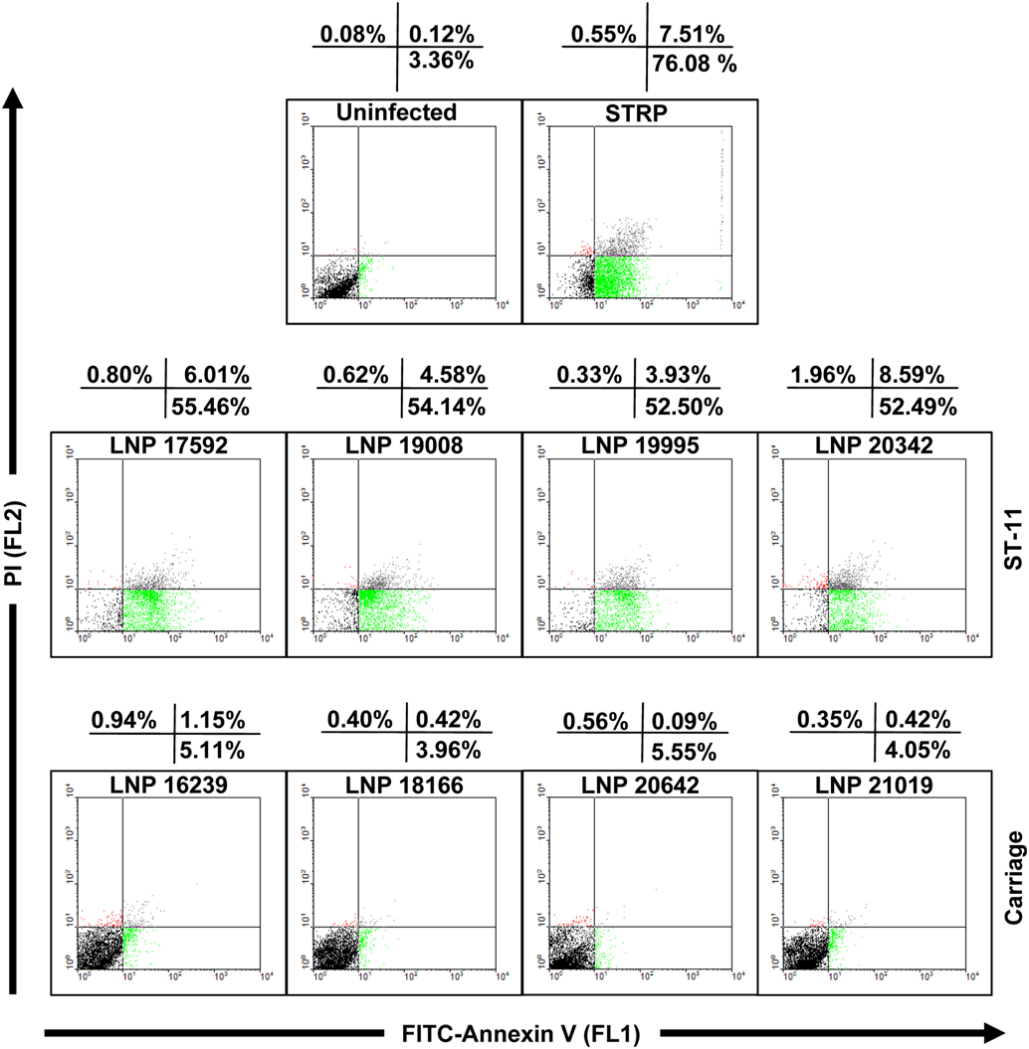
Flow cytometric analysis of Hec-1-B epithelial cells following meningococcal infection. Confluent Hec-1-B cell monolayers were infected with the indicated pathogenic ST-11 or carriage isolates for 9 h or left uninfected (control). A positive control for apoptosis, cells were treated for the same period with 1 µM STRP. After washing and Annexin V– and PI-staining, cells were analyzed by flow cytometry. Outset numbers in the top of each plot, show percentages of cells in the quadrant. Early apoptotic cells (Annexin V^+^/PI^−^, green population), dead cells (Annexin V^−^/PI^+^, red population) and late apoptotic/necrotic cells (Annexin V^+^/PI^+^, grey population). Data are representative of at least three independent experiments with similar results.

**Figure 2:**
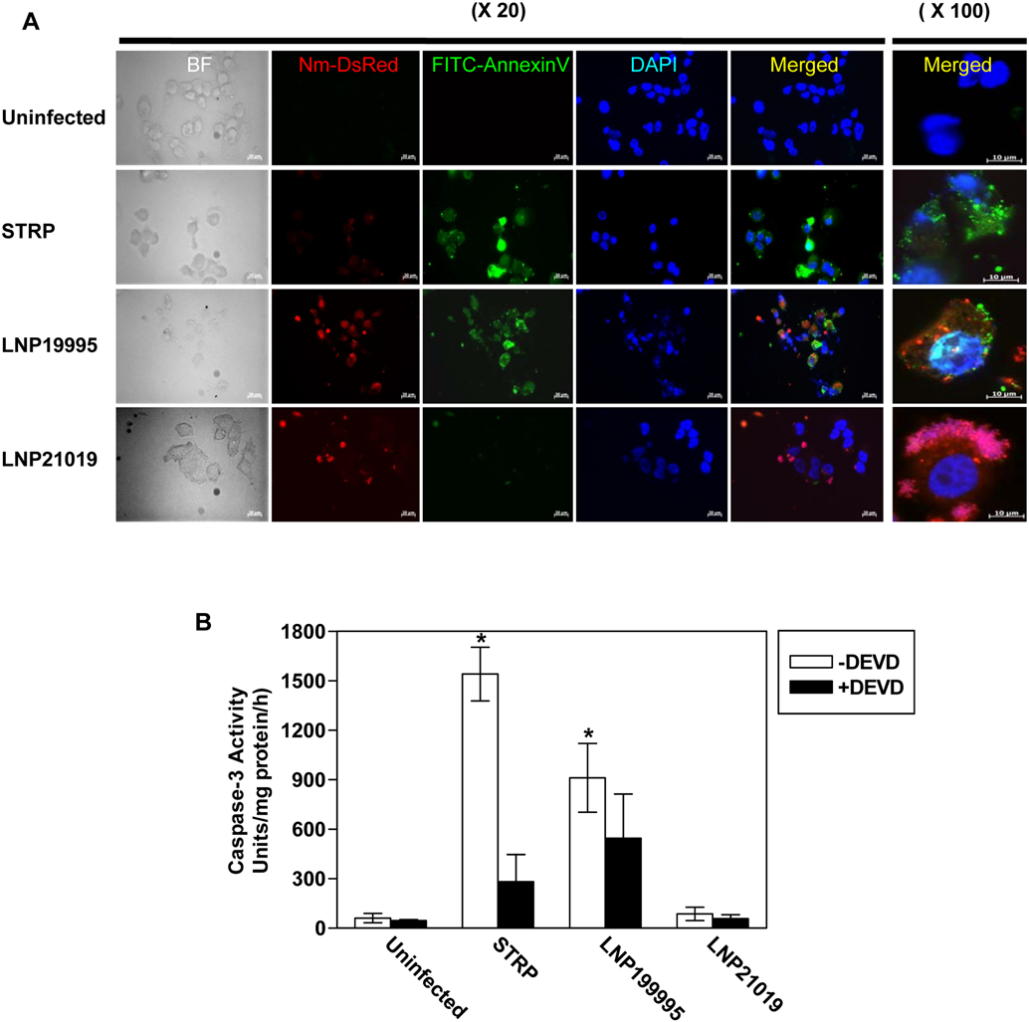
Analysis of apoptotic Hec-1-B cells by immunofluorescence microscopy and caspase-3 activity. (A) Hec-1-B epithelial cells were infected with the pathogenic isolate LNP19995 (Red) or the carriage isolate LNP21019 (Red). As controls, cells were left uninfected (negative control) or treated with STRP (positive control). Nine hours later, cells were fixed and stained with FITC-Annexin V (green) and DAPI (nuclei staining, blue) and observed under fluorescence microscope. Left panels represent images taken with low magnification (×20) and right panel are merged images taken with high magnification (×100). Green arrows indicate Annexin V positive cells. Red arrowheads indicate bacteria. BF: Bright field. Scale bars 20 µm and 10 µm for magnifications ×20 and ×100, respectively are shown. (B) The pathogenic isolate LNP19995 but not the carriage isolate LNP21019 activates Caspases-3 in Hec-1-B epithelial cells. Cells were infected with either isolate, treated with STRP (positive control) or left untreated (negative control). Cells were harvested 9 h post-infection, lysed and the caspase-3 activity was determined. The specificity of activity was monitored using caspase-3 specific inhibitor DEVD added 2 h prior to infection. The activity is expressed as arbitrary fluorescence units/mg protein/h. All samples were run in triplicate in each experiment. The results shown are representative of three independent experiments, and are expressed as mean±SD (* indicates *P*<0.001).

We selected ten pathogenic meningococcal isolates of the sequence type ST-11 (ST-11/ET-37 complex) that belong to different serogroups (B, C, and W135). Eight carriage isolates of different serogroups belonging to different clonal complexes were also included (Table 1). We first explored the cytopathic effect of these isolates to Hec-1-B epithelial cells. All disease isolates were cytopathic to Hec-1-B cells particularly after 9 h of infection, regardless their serogroups. In contrast, none of the carriage isolates tested was cytopathic (Table S1). This cytopathic effect was mainly due to induction of apoptosis in infected cells. Indeed, all the ST-11 pathogenic isolates significantly induced apoptosis in infected cells as estimated by FITC-conjugated Annexin V staining and flow cytometry (FACS) analysis (Figure 1 and Table S1). However, FITC-conjugated Annexin V staining in cells infected with carriage isolates was comparable to that of uninfected control cells. All the eighteen tested isolates grew similarly in the medium of infection (data not shown). This excludes a possible effect of differential bacterial growth on apoptosis. The induction of apoptosis was further confirmed by fluorescence microscopy. Hec-1-B cells were infected with the pathogenic isolate LNP19995 or the carriage isolate LNP21019, both expressing the red fluorescent protein DsRed, and were stained with FITC-Annexin V 9 h post-infection. As a positive control, cells were treated with 1 µM staurosporine (STRP). We found that similarly to STRP, LNP19995 efficiently induced cells to become apoptotic as revealed by FITC-Annexin V staining (Figure 2A). In contrast, Hec-1-B cells infected with the carriage isolate LNP21019 were resistant to staining with Annexin V after the same period of infection (Figure 2A). To further confirm that this alteration was due to apoptotic cell death, a number of apoptosis-specific assays were performed. Caspase-3 activity was detected in cell lysates of Hec-1-B infected with the ST-11 pathogenic isolates. This activity was blocked by the caspase-3-specific inhibitor DEVD (*N*-acetyl-Asp-Glu-Val-Asp). In contrast, a basal level of caspase-3 activity similar to uninfected cells was observed in cells infected with the non-cytopathic isolates (Figure 2B and Table S1). Further evidence of apoptosis was provided by the distinct presence of DNA laddering effect when genomic DNA was resolved by agarose gel electrophoresis. This laddering effect was absent in cells infected with carriage isolates as in untreated control cells (Figure S1). Other epithelial cell lines behave similar to Hec-1-B cells. Indeed, the pathogenic isolate LNP19995 but not the carriage isolate LNP21019, was able to induce apoptosis in A549 and HEp-2 human epithelial cell lines as revealed by FITC-Annexin V staining (Figure S2). These results suggest that the apoptotic effect of the pathogenic isolates towards infected cells is not only restricted to Hec-1-B epithelial cell line. Taken together, our data suggest that pathogenic meningococcal ST-11 isolates induced apoptosis as a major cytopathic mechanism to infected epithelial cells.

**Table 1:** *N. meningitidis* isolates used in this study and their characteristics.

	Isolate	Clonal Complex	Phenotype	Isolation Site	Adhesion (%)	Invasion (%)
Invasive	LNP13143	ST-11	W135: 2a: P1.5, 2	CSF	29.0±6.8	1.2±0.4
	LNP17592	ST-11	W135:2a: P1.5, 2	Blood	33.0±7.7	2.8±0.5
	LNP19008	ST-11	C: 2a : P1.5, 2	Blood	34.0±13.0	9.1±1.3
	LNP19995	ST-11	W135: 2a: P1.5, 2	CSF	32.0±8.6	4.8±0.1
	LNP20342	ST-11	B: 2a: P1-5, 2	CSF	33.0±0.7	2.6±0.8
	LNP20553	ST-11	C: 2a: P1.5	CSF	25.0±3.7	2.8±1.9
	LNP21515	ST-11	C: 2a: P1.5	CSF	32.0±3.4	4.8±1.0
	LNP21678	ST-11	C: 2a: P1.5	Blood	43.0±8.8	12.4±1.5
	LNP21996	ST-11	B: 2a: P1. 5	Blood	41.0±13.0	15.0±1.2
	LNP24198	ST-11	C: 2a: P1.7,1	Blood	40.5±2.1	4.6±0.8
Carriage	LNP16239	NA(1) (ST-1117)	B: NT(2): NST(3)	Expectoration	4.0±7.0	2.2±1.4
	LNP10820	ST-5	A: 4: P1.9	Expectoration	31.0±9.0	5.5±2.3
	LNP18166	ST-22	W135: NT: NST	Expectoration	37.0±3.0	4.1±0.2
	LNP1288	ST-32	B: 14: P17. 16	Nasopharynx	31.0±9.0	5.5±2.3
	LNP1934	ST-32	B: 14: P17. 16	Nasopharynx	10.0±2.1	2.2±1.4
	LNP3503	ST-32	B: 14: P1. 7, 16	Nasopharynx	6.0±2.3	3.9±1.1
	LNP21019	ST-35	B: NT:P1.14	Expectoration	34.0±11.2	6.8±3.6
	LNP20642	ST-334	C: NT: NST	Expectoration	6.0±1.0	6.7±2.5

Mutant strains	Characteristics
**NM0401**	Isogenic mutant of the pathogenic isolate LNP19995, deficient in PorB expression (*porB::erm*), EryR
**NM0705**	Isogenic mutant of the pathogenic isolate LNP19995, deficient in LOS and PorB production (*lpxA::aph3'/porB::erm*), KanR EryR
**NM0706**	Non-piliated isogenic mutant of the pathogenic isolate LNP19995, deficient in pilin (*pilE::aph3'*), KanR
**NM0707**	Non-capsulated isogenic mutant of the pathogenic isolate LNP19995 (*ctrA::aph3'*), KanR
**Z0305**	Isogenic mutant of the pathogenic isolate LNP19995, deficient in LOS production (*lpxA::aph3'*), KanR
**NM0813**	Non-capsulated isogenic mutant of the carriage isolate LNP21019 (*ctrA::aph3'*), KanR

(1) NA: not assigned to major clonal complex.

(2) NT: non-typeable.

(3) NST: non-subtypeable.

### Meningococcal ST-11 isolate requires cell adhesion, but not invasion, to induce efficient apoptosis in human epithelial cells

**Figure 3:**
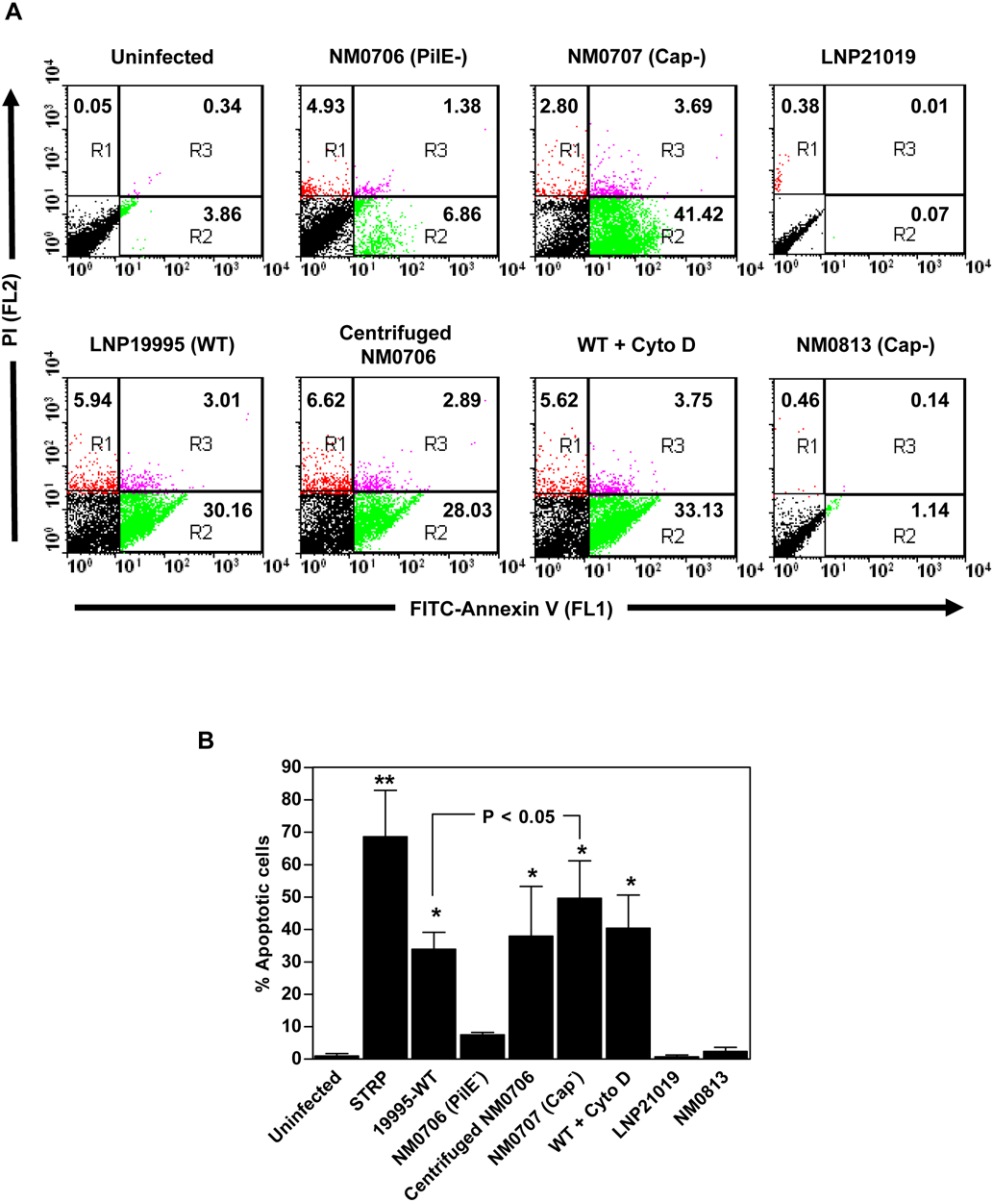
Effect of pili and capsule on apoptosis. (A) Hec-1-B epithelial cells were infected for 9 h with the pathogenic isolate LNP19995 (WT), or the isogenic mutants NM0706 (non-piliated) and NM0707 (unencapsulated), the non-pathogenic isolate LNP21019, or the unencapsulated isogenic mutant NM0813. In some experiments, the non piliated mutant NM0706 was directly centrifuged on cells or cells were treated with 10 µM cytochalasin D prior to infection with the WT strain. Cells were harvested, stained with FITC-Annexin V and PI, and submitted to FACS analysis. Inset numbers indicate the percentage of apoptotic population (green population). (B) Quantification of the results shown in A from three individual experiments. The data in (A) are from one of three independent experiments that yielded similar results and values in (B) are the mean±SD.

Isolates used in this study (both pathogenic and carriage isolates) are piliated and capsulated as evaluated by specific sera against pili and capsule (data not shown). We therefore explored the impact of these structures on the induction of apoptosis. All tested pathogenic isolates were adherent to Hec-1-B cells (Table 1). However, carriage isolates were heterogeneous in their adhesiveness to Hec-1-B epithelial cells. These observations suggest that adhesion could be necessary but insufficient to establish a cytopathic effect. We next compared induction of apoptosis in human Hec-1-B epithelial cells upon infection with the wild-type pro-apoptotic isolate LNP19995 as well as its isogenic pilus-deficient mutant NM0706, using flow cytometry following Annexin V staining. While the pilus-deficient mutant failed to induce significant apoptosis, wild-type LNP19995 triggered 5 fold increase of phosphatidylserine asymmetry breakdown as detected by an increase in FITC-Annexin V binding within 9 h of infection. Centrifugation of bacteria to infected cells conferred apoptotic properties to NM0706 strain (Figure 3A and 3B), suggesting that cell contact rather than pili expression is necessary to induce apoptosis. Moreover, no difference in the induction of apoptosis was observed in the presence of 10 µM cytochalasin D that blocks bacterial invasion to Hec-1-B cells, suggesting that invasion is not involved in this process. An isogenic capsule deficient mutant (NM0707) was also compared to its parental strain LNP19995 and showed a higher level of induction of apoptosis in Hec-1-B cells than the parental strain (Figure 3A and 3B). In contrast, the capsule-deficient isogenic mutant of the carriage isolate LNP21019 did not show any significant change in the apoptotic level compared to the parental strain (Figure 3A). None of these tested mutants showed growth defect when compared to their parental strains, excluding the possibility of differential apoptosis due to differential growth rate (data not shown). These results strongly suggest that capsule and pili expression is not directly correlated with induction of apoptosis.

### LOS and PorB synergistically promote apoptosis of epithelial cells by the meningococcal pathogenic isolate

**Figure 4:**
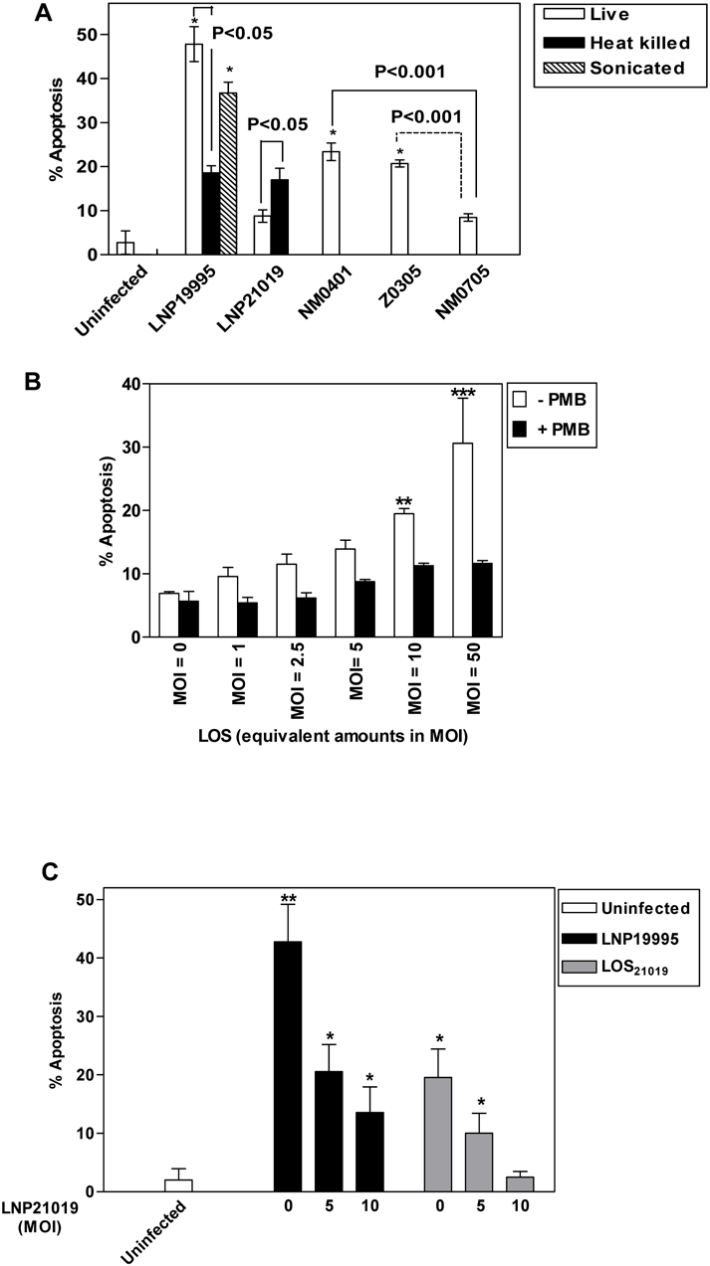
LOS and PorB are required for induction of apoptotic cell death by the pathogenic isolate LNP19995. (A) Hec-1-B cells were infected with live or killed LNP19995 and LNP21019 or the isogenic mutants of the isolate LNP19995 defective in PorB (NM0401), LOS (Z0305) or both PorB and LOS (NM0705). Apoptosis was measured by FITC-Annexin V/PI double staining after 9 h of infection. Uninfected cells were used as negative control. The data are representative of three different experiments, and error bars represent the standard deviations of triplicate samples. (B) Purified LOS induces apoptosis in a dose-dependent manner. Cells were treated at different concentrations with LOS for 9 h in absence (−) or presence (+) of 1 µM polymixin B (PMB). The percentage of apoptotic cells was determined using the ApoPercentage kit. Untreated (MOI = 0) cells served as the negative control. Numbers in the bottom of histograms indicate the MOIs corresponding to the amount of LOS used. (C) The non-pathogenic isolate LNP21019 actively inhibits the apoptosis induced by LOS or the pathogenic isolate LNP19995. Cells infected with LNP19995 at MOI 10 (dark bars) or equivalent amounts of LOS purified from LNP21019 (grey bars) were co-challenged with an increasing MOI of the isolate LNP21019. After 9 h, cells were harvested and stained with FITC-Annexin V and PI. Numbers under histograms indicate LNP21019 to cell ratio. The data are the mean±SD of three separate experiments (** indicates *P*<0.01 and * indicates *P*<0.05).

We next showed that sonicated extracts of the pathogenic isolate LNP19995 was able to induce apoptosis to a similar level to that observed with live LNP19995 bacteria. In addition, heat-killed bacteria (60°C for 30 min) of the isolate LNP19995 were still able to induce apoptosis in Hec-1-B cells although to a significant lesser extent (Figure 4A). These results suggest that bacterial growth rate does not affect the induction of apoptosis. Moreover, both proteinaceous and non proteinaceous bacterial factors are involved in inducing apoptosis. Indeed, both the isogenic LOS-defective mutant Z0305 and the isogenic PorB-defective mutant NM0401, showed reduced levels of induction of apoptosis that were similar to that induced by heat-killed parental wild-type bacteria (Figure 4A). Infection with the double knock out mutant NM0705, defective in both LOS and PorB production, abrogated apoptosis to a level comparable to uninfected control cells (Figure 4A). Both purified LOS and PorB from the pathogenic isolate LNP19995 were able to induce apoptosis in Hec-1-B cells in a dose-dependent manner. The effect of LOS was abrogated by treatment of cells with the endotoxin drug inhibitor polymixin B (Figure 4B and data not shown). Surprisingly, purified LOS from the carriage isolate LNP21019 induced apoptosis to a similar extent to that purified from the pathogenic isolate LNP19995 (data not shown). Moreover, heat-killed bacteria of the carriage isolate also induced apoptosis (Figure 4A). Taken together, these results suggest that the pathogenic ST-11 isolate LNP19995 induces apoptosis, at least in part, in LOS and PorB-dependent manner. This process was actively suppressed by the carriage isolate. To further confirm this hypothesis, we designed a “functional” complementation using co-infection experiments. Hec-1-B cells were infected with the pathogenic isolate LNP19995 or treated with purified LOS from the isolate LNP21019 in presence of increasing numbers of the non-pathogenic isolate LNP21019. Figure 4C clearly shows that apoptosis induced by the pathogenic isolate LNP19995 or LOS treatment decreased gradually by the co-infection of Hec-1-B cells with the non-pathogenic isolate LNP21019 in dose-dependent manner.

### The ST-11 invasive isolate LNP19995 induced apoptosis through TNF-α–dependent and TNF-α–independent pathways

**Figure 5:**
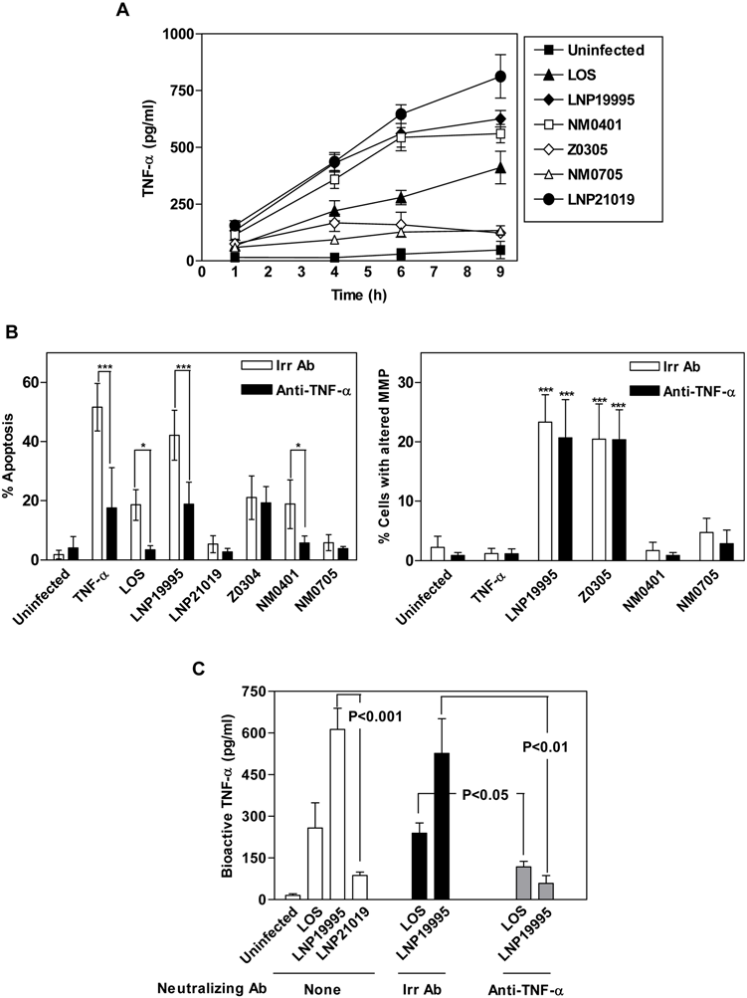
Analysis of TNF-α secretion and bioactivity. (A) Quantitation of TNF-α in culture supernatants of Hec-1-B cells infected with the indicated strains. Cells were infected with the indicated strains, treated with LOS, or left untreated, and culture supernatants were collected at 9 h post-infection and assayed for TNF-α secretion by ELISA. Shown are mean±SD of two representative experiments. (B) *Left panel:* Competitive inhibition of apoptosis by an anti–TNF-α neutralizing antibody. Hec-1-B cells were infected, or treated with TNF-α for 9 h in presence of either an anti–TNF-α neutralizing antibody (closed bars) or an isotype-matched irrelevant antibody (Irr Ab, open bars). Cells were harvested and stained with FITC-Annexin V and PI before FACS analysis. Data are the mean±SD of three independent experiments. *Right panel*: LNP19995 affects the mitochondrial membrane potential (MMP) in PorB-dependent, TNF-α-independent manner. Cells were infected as described previously. After 9 h of incubation, cells were harvested and the mitochondrial membrane potential damage was performed using DiIC1_(5)_ dye staining and FACS analysis. Acquisition was performed on 10,000 events. Measurements are representative of three independent experiments. (C) The non-pathogenic carriage isolate LNP21019 altered TNF-α bioactivity. Culture supernatants from Hec-1-B cells at 9 h after infection with LNP19995, LNP21019 or LOS treatment, were assayed for TNF-α bioactivity by measuring cytotoxicity for L929 mouse fibroblasts. Dilutions of recombinant human TNF-α (rhTNF-α) were used as internal standard. The specificity of TNF-α bioactivity was monitored by incubation of Hec-1-B cultures in presence of specific anti–TNF-α neutralizing or irrelevant (Irr) antibodies. Data are mean±SD from three independent experiments. The *P* values are shown.

Meningococcal LOS is a potent inducer of TNF-α [26]. TNF-α is also known to trigger apoptosis of a diverse range of cells *in vitro* [27]. Surprisingly, both the pathogenic isolate LNP19995 and the carriage isolate LNP21019 as well as purified LOS, were shown to induce secretion of TNF-α (Figure 5A). However, only the pathogenic isolate LNP19995 and purified LOS were able to induce apoptosis in Hec-1-B cells. This induction was dramatically reduced using anti–TNF-α neutralizing antibody (Figure 5B, left panel). Similar results were obtained using A549 and HEp-2 epithelial cell lines (Figure S2). However, induction of apoptosis by TNF-α in these cell lines required cyclohexemide (CHX) treatment, an inhibitor of eukaryotic protein synthesis (Figure S2). The decrease of apoptosis in Hec-1-B cells challenged with the LOS-devoid mutant Z0305 correlated with the decrease of released TNF-α (Figure 5A). The remaining apoptotic activity of this mutant was insensitive to anti–TNF-α neutralizing antibody (Figure 5B, left panel). In contrast, the residual induction of apoptosis in Hec-1-B cells when infected by the PorB-deficient mutant NM0401 (Figure 5A) was abolished in presence of anti–TNF-α neutralizing antibody compared to cells treated with an irrelevant antibody (Figure 5B, left panel). We further showed that infection of Hec-1-B cells with the wild-type isolate LNP19995 or the LOS-deficient mutant Z0305 was associated with an alteration of the mitochondrial membrane potential (MMP). This alteration was not affected by an anti–TNF-α antibody. Moreover, this alteration was neither observed when infection was performed using PorB-deficient mutants (NM0401 and NM0705) nor upon TNF-α treatment. (Figure 5B, right panel). Taken together, these results suggest that ST-11 pathogenic isolates induce apoptosis through both an extrinsic pathway involving LOS induction of TNF-α and an intrinsic pathway involving PorB.

To understand why the non-pathogenic isolate LNP21019 did not induce apoptosis despite the similar release of TNF-α as the pathogenic isolate LNP19995, the bioactivity of secreted TNF-α was evaluated using the cell line L929 as described in Materials and Methods. Bioactive TNF-α was only detected when Hec-1-B cells were infected by the pathogenic isolate LNP19995 and purified LOS and was specifically reduced by anti–TNF-α neutralizing antibody (Figure 5C).

### Bioactive TNF-α–induced apoptosis in epithelial cells infected by ST-11 meningococcal isolate through TNF-RI receptor signaling

**Figure 6:**
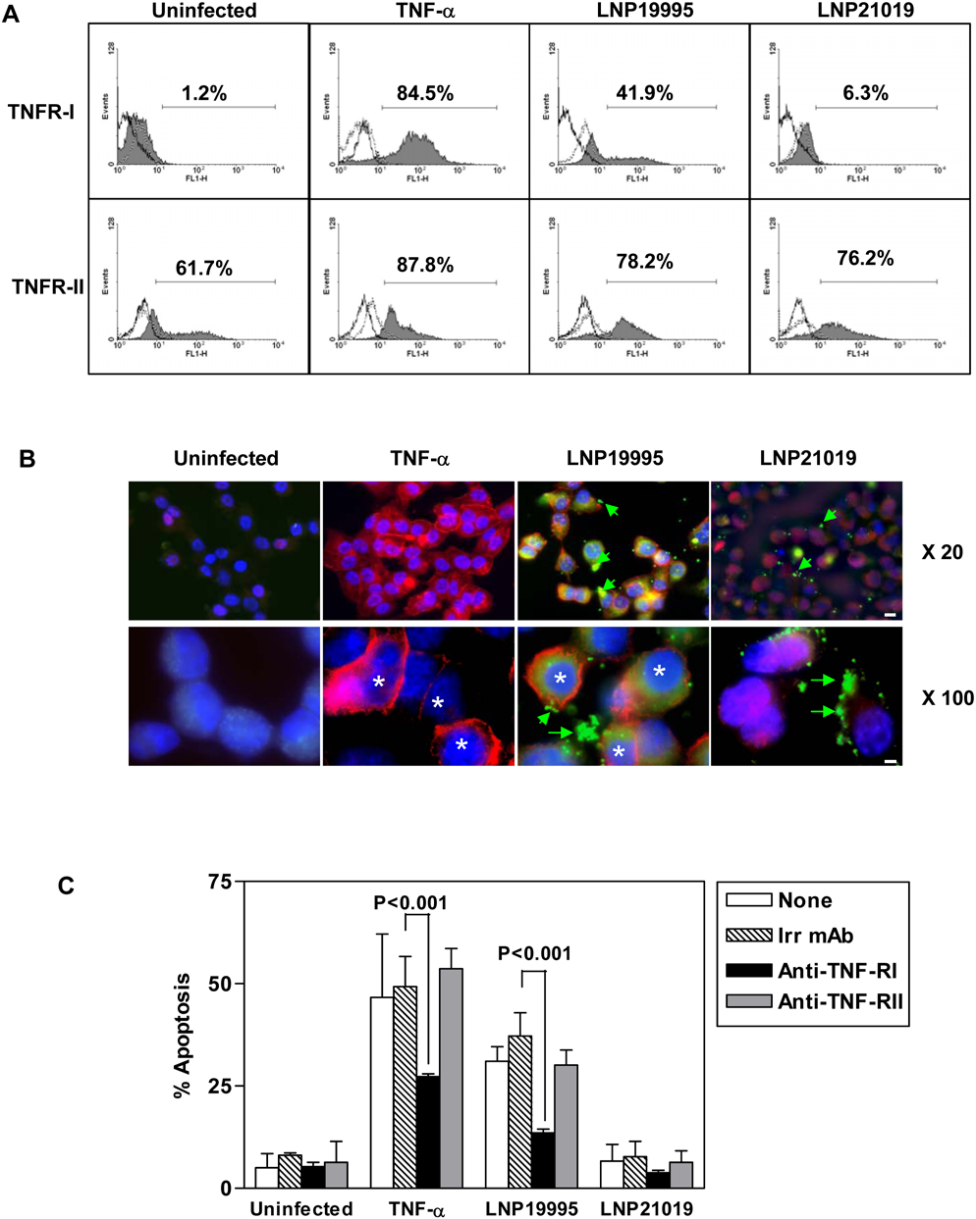
Differential cell surface expression of TNF-RI upon infection with pathogenic and carriage isolates. (A) The non-pathogenic carriage isolate specifically alters surface expression of TNF-RI. Hec-1-B cells were either left untreated in culture medium alone, treated with TNF-α, or incubated with bacteria for 9 h. Cells were harvested and incubated with anti-TNF-RI or anti-TNF-RII specific antibodies, followed by labeling with FITC-conjugated secondary Abs. Samples were washed and fixed in 2.5% paraformaldehyde before FACS analysis under non-permeabilizing conditions. Results are expressed as the percentages of positive cells stained with the specific antibody relative to irrelevant antibody derived from 10,000 events and are indicated in each panel. *The solid open histograms*, unstained cells; *dashed histograms*, cells stained with an irrelevant isotype-matched antibody; *the grey filled histograms*, cells stained with specific antibody. The data shown are representative of three separate experiments. (B) Immunofluorescence analysis of TNF-RI surface expression. Cells adhering to tissue culture coverslips were incubated with TNF-α or infected with GFP- bacteria (green) for 9 h. Cells were then fixed and stained for TNF-RI (Red) and with DAPI to visualize nuclei (blue). Labeled cells were examined under a digital immunofluorescence microscope using low (×20, upper panel) or high (×100, lower panel) magnifications. Green arrows and asterisks indicate bacteria and nuclei, respectively. Scale Bars are 20 µm and 10 µm for magnifications ×20 and ×100, respectively. (C) Competitive inhibition of apoptosis by mAb to TNF-RI. Hec-1-B cells were infected or treated with TNF-α for 9 h in absence or presence of mAbs to either TNF-RI or TNF-RII or an irrelevant antibody at a final concentration of 20 µg/ml. Cells were then assayed for apoptosis using ApoPercentage kit. Values shown are the mean±SD of three independent experiments.

We therefore explored the mechanism leading to alteration of TNF-α bioactivity upon infection with the carriage isolate. TNF-α induces apoptosis through signaling with its death domain-containing receptor TNF-RI [28]. We next showed, using FACS analysis, that after 9 h of bacterial infection a sustained increase in TNF-RI staining was only observed at the surface of Hec-1-B cells infected with LNP19995 but not at the surface of Hec-1-B cells infected with LNP21019 (Figure 6A). Surface expression of TNF-RII, another receptor belonging to TNF-α receptor superfamily but lacking the death domain, remained unaffected (Figure 6A bottom panel). We complemented our flow cytometry studies by immunofluorescence microscopy to visualize the altered surface expression of TNF-RI in Hec-1-B cells infected with LNP21019. Monolayers of Hec-1-B cells were infected with GFP-expressing LNP21019 or LNP19995 and were stained for TNF-RI at 9 h post-infection. As TNF-α treatment, cells infected with LNP19995 exhibited increased surface staining of TNF-RI compared to uninfected cells. In contrast, cells infected with LNP21019 showed low level surface staining pattern similar to uninfected cells (Figure 6B).

Pre-incubation of Hec-1-B cells with anti-TNF-RI monoclonal antibody (mAb), but not with anti-TNF-RII, significantly reduced apoptosis induced by the isolate LNP19995 and TNF-α compared to an irrelevant antibody (∼23% decrease). No changes were observed after infection with the carriage isolate LNP21019 (Figure 6C). Under all tested conditions, antibody treatment did not affect the adhesion levels of meningococcal isolates (data not shown). Taken together, these data suggest that TNF-RI may be involved in epithelial cell death induced by pathogenic isolates.

### The meningococcal carriage isolate, but not the pathogenic ST-11 isolate, increased shedding of TNF-RI receptor

**Figure 7:**
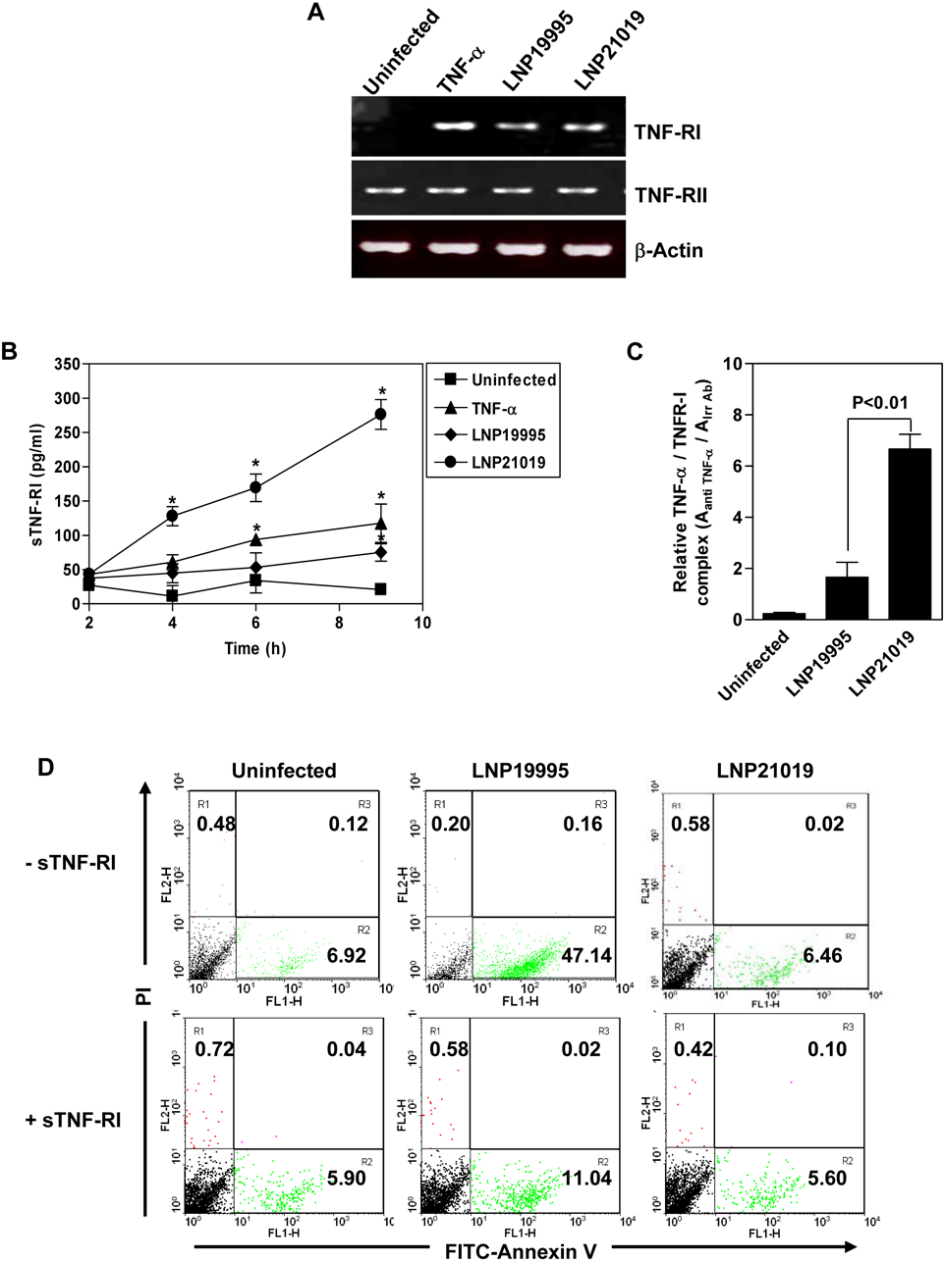
Analysis of transcription and shedding of TNF-RI. (A) RT-PCR analysis of *TNFRI* and *TNFRII* expression. Hec-1-B cells were incubated in medium alone (uninfected), stimulated with TNF-α or infected with LNP19995 (pathogenic) or LNP21019 (carriage) isolates for 9 h. Total RNAs were isolated and equal amounts were analyzed by RT-PCR using TNF-RI, TNF-RII and β-actin specific primers. The data shown are from one of two independent experiments that yielded similar results. (B) Levels of soluble TNF-RI (sTNF-RI) in supernatants from uninfected or infected cells. Supernatants were cleared from bacteria and levels of sTNF-RI were determined by ELISA as described in Materials and Methods. Shown are mean±SD of triplicate values from one representative experiment of two. (C) Concentration of TNF-α bound to sTNF-RI (representative for TNF-α-sTNF-RI complexes) in cultures of Hec-1-B uninfected (control) or cells infected with LNP19995 or LNP21019. TNF-α-sTNF-RI complexes were assayed by mixed ELISA (see Materials and Methods) of supernatants harvested 9 h after infection. The data presented are mean±SD of three independent experiments. (D) Soluble TNF-RI (sTNF-RI) inhibits apoptosis induced by LNP19995 isolate. Cells were incubated alone or infected with either LNP19995 or LNP21019 for 9 h in the presence or in the absence of 250 pg/ml of sTNF-RI. After staining with FITC-Annexin V and PI, apoptotic cells were analyzed by FACS. Inset numbers represent the percentage of each population in the quadrants. Data are representative of two independent experiments which yielded similar results.

The reduction of TNF-RI cell surface expression by the carriage isolate did not seem to result from transcriptional down-regulation of its encoding gene *TNFRI*. RT-PCR analysis showed that *TNFRI* was up-regulated upon TNF-α stimulation compared to uninfected cells. Surprisingly, bacterial challenge brought about marked and comparable up-regulation in mRNA levels (Figure 7A). Neither the expression of TNF-RII encoding gene (*TNFRII*) nor the level of expression of β-actin encoding gene (internal control) showed any alteration under the tested conditions (Figure 7A). Moreover, FACS and Western blot analysis in permeabilized cells after 9 h of infection further showed that intracellular amounts of TNF-RI increased similarly in cells infected with each isolate compared to uninfected cells (Figure S3). To explain the low level of surface expression of TNF-RI in cells infected with the carriage isolate, we monitored the level of soluble receptor (sTNF-RI) formation. Indeed, TNF-RI is known to decrease from the cell surface by shedding of the extracellular domain into surrounding environment leading to formation of sTNF-RI [29]–[31]. Supernatants from Hec-1-B infected cell cultures were collected 2, 4, 6, and 9 h post-infection and assayed for sTNF-RI by Enzyme-Linked ImmunoSorbent Assay (ELISA). While low levels of sTNF-RI were detected in uninfected cells over time, the level of sTNF-RI strongly increased in a time dependent manner in cells infected with LNP21019 and was 3 times higher than in cells infected with LNP19995 at 9 h post-infection (Figure 7B). The difference in the sTNF-RI levels was statistically significant at 6 and 9 h after infection (*n* = 3, *P*<0.05). Levels of sTNF-RII measured by ELISA did not significantly change following infection (data not shown).

The presence of TNF-α bound to sTNF-RI in supernatants of cell cultures infected with LNP21019 was examined by a mixed ELISA. The TNF-α/sTNF-RI complexes were captured by immobilized antibodies to sTNF-RI and quantified by using antibodies to TNF-α. Detectable levels of TNF-α/sTNF-RI complexes were found in cultures of Hec-1-B infected with both *N. meningitidis* isolates and were not found in uninfected cells. The level of complexes in the supernatant of Hec-1-B cells infected with the carriage isolate LNP21019 was about 4 times higher than that in the supernatant of Hec-1-B cells infected with the pathogenic isolate LNP19995 (Figure 7C). Taken together, these results strongly suggest that the carriage isolate LNP21019 in contrast to the ST-11 pathogenic isolate LNP19995 provoked significant shedding of TNF-RI that seems to chelate TNF-α in the cellular environment. To further establish a causal relationship between the shedding of TNF-RI and the protection from TNF-α-induced apoptosis, Hec-1-B cells were infected with the pathogenic isolate LNP19995 in presence of 250 pg/ml of sTNF-RI (which is similar to the level of sTNF-RI shedded upon infection with the non-pathogenic isolate LNP21019). Figure 7D showed that treatment of cells with sTNF-RI led to decrease of apoptosis induced by the pathogenic isolate LNP19995. These results strongly suggest that the release of sTNF-RI is a potential mechanism of protection against TNF-α-induced cell death.

## Discussion

Asymptomatic carriage of *N. meningitidis* contrasts sharply with invasive infections that are characterized by severe symptoms and a major inflammatory response particularly cytokine production and coagulopathy [32],[33]. Host susceptibility is known to impact on meningococcal disease (for review see [34],[35]). Most likely, the co-evolution between this exclusive human bacterium and its host derived diversity in both host and bacteria [36]. Moreover, MLST analysis revealed that a limited number of “hyper-invasive lineages” are most frequently associated with meningococcal disease [37],[38] and that isolates belonging to these lineages, particularly ST-11 isolates, are under-represented in healthy carriers [22]–[24]. In a previous study we showed a positive correlation between virulence in mice and proapoptotic effects to epithelial cell line and isolates of the hyper-invasive lineage ST-11 [25]. We further confirmed here that ST-11 isolates from invasive meningococcal infection are responsible for constant induction of apoptosis in Hec-1-B epithelial cell model, in contrast to isolates from healthy carriers. Moreover, we have previously reported that pathogenic isolates of other clonal complexes (such as the clonal complex ST-32) variably induced apoptosis and that correlated with heterogeneous virulence in mice and higher diversity of isolates compared to ST-11 isolates [25]. Indeed, well established laboratory isolates such as the isolates MC58 and H44/76 (both belonging to the clonal complex ST-32) were unable to induce apoptosis in Hec-1-B cells (data not shown).

A growing number of studies have shown that apoptosis can be modulated (inhibited or promoted) by bacteria and protozoan parasites (for a review see [10]–[13]). Recently, Schubert *et al.* [15] reported up-regulation of apoptosis-related genes (*bad*, *bak*, *asp*, and immediate-early response gene 1) in meningococcal infected cells. Further analyses confirmed that cells displayed several hallmarks of apoptosis in response to meningococcal infection, namely, phosphatidylserine translocation and activation of caspase-3 and AMP-activated protein kinase.

Meningococcal adhesion but not invasion seems to be necessary for the induction of apoptosis. The initial contact with the host cells is brought by pili, where a strong association of the pathogen to the cell takes place [39]. Our results indicate that adhesion contributes to induce apoptosis of cells infected with pathogenic isolates. This could occur through increasing local delivery of bacterial factors (such as LOS and PorB) leading to apoptosis of infected cells. In favor of this explanation is the fact that greater and earlier apoptosis was observed in response to the capsule-deficient mutant than in response to the wild-type strain, most likely through unmasking bacterial surface. Moreover, the abrogation of apoptosis in cells infected with the mutant strain deficient in both PorB and LOS production is also in favor of this hypothesis. In *Pseudomonas aeruginosa*, pili-producing strains were essential in initiating apoptosis of human Chang conjunctiva cells [40] and piliated enteropathogenic *Escherichia coli* significantly enhanced apoptotic cell death in HeLa and Caco-2 cell lines, compared to non-piliated mutants [41].

In the current study, we have shown that both meningococcal LOS and PorB act independently but synergistically to induce apoptosis in several human cell lines. Conflicting results on the role of the major outer membrane porin, PorB, in the induction of apoptosis in pathogenic *Neisseria* (*N. gonorrhoeae* and *N. meningitidis*) have been reported [16],[17],[20]. Similar to our experimental conditions, PorB-related apoptosis has been reported in absence of serum [17]. Absence of serum did not modify the LOS effect as all cell lines tested in this study express membrane CD14 and TLR4 ([42],[43] and data not shown). LOS signaling is dependent on TLR4 [44]–[46], while meningococcal PorB has been described as the ligand of TLR2 [47],[48]. Although signaling through both receptors lead to TNF-α release, our data suggest that additional PorB-induced apoptosis to be independent from secretion of TNF-α and may occur through the intrinsic pathway by alteration of the mitochondrial membrane permeability [16],[17].

The role of endotoxin of Gram negative bacteria (including Neisserial LOS) in cytotopathic effects has been documented [49],[50]. The severity of meningococcal disease is thought to be linked to the degree of the inflammatory response induced during invasive infection [1],[51]. As a potent inducer of pro-inflammatory cytokine response, meningococcal LOS seems to play a key role in inducing apoptosis through TNF-α [52],[53]. In the current study, we have shown that invasive isolates of *N. meningitidis* induced the secretion of TNF-α and apoptosis in Hec-1-B epithelial cells that could be abrogated by the addition of anti–TNF-α neutralizing antibodies. A relationship between TNF-α secretion and apoptosis in the fallopian tube has also been suggested by studies using a mouse model of infection with a *Chlamydia trachomatis* mouse-specific pneumonitis strain. Infection with this pathogen leads to a large increase in apoptotic cells in murine oviducts, but treatment with anti–TNF-α antibodies leads to a significant decrease in the level of apoptosis in the upper genital tract [54].

Induction of TNF-α and subsequent apoptosis at the portal of entry of *N. meningitidis* (the nasopharynx) could enhance the invasiveness of pathogenic isolates and hence promote the hyper-invasiveness of isolates belonging to the clonal complex ST-11. However, the origin of locally induced TNF-α remains to be determined. It might be produced by resident macrophages and/or airway epithelial cells. It has been shown that TNF-α levels were significantly higher in children with demonstrable *H. influenzae* growth in nasopharyngeal cultures than in culture-negative children [55].

An important mechanism for inducing apoptosis is the activation of the death receptor pathway by extracellular death-inducing ligand TNF-α, which binds to the cognate cell surface receptor TNF-RI [56]. A major response to infectious disease is a cytotopathic effect resulting from activation of this pathway, and the production of TNF-α has been shown to correlate with a cytopathic effect in infected cells [19],[57]. Indeed, we were able to demonstrate that neutralization of TNF-RI with a specific antibody as well as pretreatment of cells with sTNF-RI provoked a decrease of apoptosis generated by the invasive meningococcal isolate. Moreover, we further showed that meningococcal carriage isolate (but not pathogenic isolate) can manipulate this pathway by increasing the shedding of TNF-RI, resulting in inactivation of TNF-α by soluble receptor-ligand complex formation. The mechanism of this enhancement is not yet clear. Shedding of TNF-RI can be mediated by TNF-α converting enzyme (TACE/ADAM 17), a metalloproteinase localized in the cytoplasmic membrane [58],[59]. A plausible explanation of the resistance to TNF-α-induced apoptosis by the carriage non-pathogenic isolates is the activation of TACE leading to generation of soluble TNF-α-binding protein that binds and inhibits TNF-α bioactivity. *Chlamydia trachomatis* and *Streptococcus pneumoniae* have been shown to reduce the display of TNF-RI at the surface of infected HEp-2 and the airway epithelial cells, respectively through activation of TACE [60]–[63]. Alternatively, an unknown TNF-RI “sheddase” could be expressed from the non-pathogenic isolates, to undergo an interaction with and cleavage of TNF-RI.

The molecular basis of the pathogenicity of *N. meningitidis* remains poorly understood. Full genome sequencing failed to reveal specific components that could determine transmissibility and colonization by carriage isolates or invasive infection by hyper-invasive isolates. The present study provides further evidence for the ability of pathogenic isolates of the ST-11 complex to exert cytopathic effects on epithelial cells by inducing apoptosis [25]. Our data strongly suggest that invasiveness of isolates of the clonal complex ST-11 positively correlates with the induction of apoptosis by these isolates. At the opposite, carriage isolates have co-evolved with human in a manner to reduce inflammatory response and apoptosis induction that may correlate with the state of asymptomatic carriage.

## Materials and Methods

### Reagents and chemicals

RPMI 1640, HBSS, and trypsin-EDTA were purchased from Invitrogen (Cergy, France). Anti-human TNF-α polyclonal antibody (Anti hTNF-α) was from MBL (Montrouge, France). Anti-TNF-RI monoclonal (mAb, Clone 16803) and anti-TNF-RII polyclonal antibodies were from R & D system (Lille, France). FITC-, Rhodamine- and HRP-conjugated secondary antibodies were from Invitrogen. Caspase 3 Assay Kit, Polymixin B and Staurosporine (STRP) were purchased from Sigma Aldrich (Lyon, France). FITC-Annexin V kit was from Immunotech (Marseille, France). ApoPercentage Apoptosis Kit was purchased from Biocolor Ltd. (Newtownabbey, Northern Ireland, UK). DiIC1_(5)_ assay kit was purchased from Molecular Probes (France).

### Bacterial strains and meningococcal characterization

Meningococcal clinical isolates in France are sent to the National Reference Centre for Meningococci (NRCM) for full determination and typing. Bacteria were grown in GCB medium with Kellogg supplements [64]. Phenotypes (serogroup: serotype: serosubtype) and MLST genotypes were determined as previously described [21]. Sequence types (ST) and clonal complexes were assigned using the *Neisseria* MLST database (http://pubmlst.org/neisseria). All *N. meningitidis* strains used in this study and their characteristics are listed in Table 1. The Pilin-deficient mutant strain NM0706 was generated by transforming the wild type strain LNP19995 with the genomic DNA of the previously described strain *pilE::aph3'* [65]. The isogenic mutants Z0305 devoid of LOS (*lpxA::aph3'*) was obtained by transforming the wild type strain LNP19995 with genomic DNA isolated from the previously described mutant strain Z0204 [38]. Isolates were selected on GCB plates supplemented with 100 µg/ml of kanamycin. To construct the non-capsulated mutant NM0707 and PorB-deficient mutant strain NM0401, open reading frame of either *ctrA* or *porB* genes were PCR-amplified from the pathogenic isolate LNP19995 genomic DNA using the primer sets CtrA-1F/CtrA-100R and PorB0-F/PorB-100R, respectively (Table 2). PCR fragments were cloned into pGEM®-T Easy (Promega), generating pGEM-CtrA and pGEM-PorB recombinant vectors, respectively. Blunt-ended PCR-generated cassettes *aph3'* (conferring resistance to kanamycin) and *erm* (conferring resistance to erythromycin) were inserted into the blunt-ended unique sites *pml*I and *Kpn*I which cut within *ctrA* and *porB* respectively, resulting into the recombinant vectors pGEM-ctrA::aph3' and pGEM-porB::erm, respectively. These recombinant vectors were separately introduced into LNP19995 and transformants were selected on kanamycin- or erythromycin-supplemented GCB agar plates, respectively. The strain NM0705 inactivated in both *porB* and *lpxA* genes was generated by transforming the genomic DNA of Z0305 strain (*lpxA::aph3'*) in the strain NM0401 (*porB::erm*). Positive clones were selected on GCB agar plates supplemented with 100 µg/ml kanamycin and 15 µg/ml erythromycin. To construct the non-capsulated mutant strain NM0813, *ctrA* was PCR-amplified from the genomic DNA of LNP21019 using the primers 0801-Fw/0801-Rev (Table 2) and cloned into pGEM-T easy plasmid. The blunt-ended *aph3'* cassette was then inserted into the *pml*I restriction site. The resulting recombinant vector was transformed into the parental strain LNP21019 and transformants were selected onto GCB agar plates supplemented with 100 µg/ml kanamycin. All knock-out mutants were confirmed by PCR and Southern blotting. To rule out the possibility that inserted cassettes may have a polar effect, RT-PCR analysis were performed to monitor the expression of genes downstream the inactivated target. The absence of pilin expression from the mutant NM0706 and the absence of capsule expression from the mutants NM0707 and NM0813 were further confirmed by immunolotting using specific polyclonal sera. Silver stained sodium dodecyl sulfate-polyacrylamide gel electrophoresis (SDS-PAGE) and ELISA were further used to confirm the absence of LOS and PorB expression from corresponding mutants.

**Table 2:** Primers used in this study.

Primer	Sequence	Characteristics
CtrA-1F	5′-CGTCACGCAGTATTATTATTGTGTGTGGAAG-3′	Coding strand of *ctrA* gene of serogroup W135
CtrA-100R	5′-CTGTTCGCGCCACTGGTAACC-3′	Non-coding strand of *ctrA* gene of serogroup W135
0801-Fw	5′-GTGTTTAAAGTGAAATTTTA-3′	Coding strand of *ctrA* gene of serogroup B
0801-Rev	5′TTAATTAGTTAAATTATTAA-3′	Non-coding strand of *ctrA* gene of serogroups B
PorB0-F	5′-TTTTCCCAGTCACGACGTTGTAATGAAAAAATCCCTGATTGCCCTGAT-3′	Coding strand of *porB* gene
PorB-100R	5′-TGTGAGCGGATAACAATTTCGAATTTGTGGCGCAG ACCGAC-3′	Non-coding strand of *porB* gene
EGFP-Fw	5′-CTG*GGATCC*TA**AGGAAG**CCCACCATGGTGAGCAA-3′	Coding strand of *egfp*, harboring *BamH*I adapter (italic) and the SD sequence (Bold)
EGFP-Rev	5′-CCC*AAGCTT*TTATCTAGATCCG-3′	Non-coding strand of EGFP gene harboring *Hind*III adapter (Italic)
Red-Fw	5′-CTG*GGATCC*TA**AGGAAG**CTCCACCATGGCCTCCTC-3′	Coding strand of DsRed gene harboring *BamH*I adapter (italic) and the SD sequence (Bold)
Red-Rev	5′-CCC*AAGCTT*TAGAGTCGCGGCCGCTACAG-3′	Non-coding strand of DsRed gene) harboring *Hind*III adapter (Italic)
PorB-3	5′-GGTGCTGAAGCACCAAGTGA-3′	5′ of *porB* promoter region
PorB-4	5′-GGCAATCAGGGATTTTTTCA-3′	3′ of *porB* promoter region
KM-6	5′-CCCAGCGAACCATTTGAGG-3′	Coding strand of kanamycin resistance cassette *aph3'*
KM-7up	5′-**GCCGTCTGAA**TGCTTTTTAGACATCTA-3′	Non-coding strand of kanamycin resistance cassette *aph3'*, with *N. meningitidis* up-take sequence (Bold)
ERAM-1	5′-GCAAACTTAAGAGTGTGTTGA-3′	Coding strand of erythromycin resistance cassette *erm*
ERAM-3	5′-AGCTT**GCCGTCTGAA**TGGGACCTCTTTAGCTTCT-3′	Non-coding strand of erythromycin resistance cassette *erm*, with up-take sequence (Bold)
TNFR1-Fw	5′-CCCCTGGCCCCAAACC-3′	Coding strand of human *TNFRI* cDNA
TNFR1-Rev	5′-CTCCCACTTCTGAAGG -3′	Non-coding strand of human *TNFRI* cDNA
TNFR2-Fw	5′-CTCTTCCAGTTGGACT-3′	Coding strand of human *TNFRII* cDNA
TNFR2-Rev	5′-AGAATCTGAGCTCCCG-3′	Non-coding strand of human *TNFRII* cDNA
Actin-Fw	5′-CACCCCGTGCTGCTGACCGAG-3′	Coding strand of human β-actin cDNA
Actin-Rev	5′-CCACACGGAGTACTTGCGCTC-3′	Non-coding strand of human β-actin cDNA

### Construction of green and red fluorescent Nm strains

The EGFP and DsRed-N1 encoding genes (ClonTech) were amplified using the primers EGFP-Fw/EGFP-Rev and Red-Fw/Red-Rev, respectively. Both forward primers harbor *BamH*I restriction site and reverse primers harbor *Hind*III restriction site as adapters to facilitate the cloning (Table 2). EGFP and DsRed fragments were subcloned into the previously described pAD3 vector [66] restricted with *BamH*I and *Hind*III, resulting in recombinant vectors pCRG-GFP and pCRG-Red, respectively. The 400 bp blunt-ended promoter region of *porB* amplified using the primers PorB3/PorB4 (Table 2) was inserted into *BamH*I site of pCRG-GFP and pCRG-Red placing both EGFP and DsRed under control of the constitutive meningococcal promoter *porB*. The resulting vectors were called pCV1 and pCV3, respectively. Blunt-ended *aph3*' cassette was then inserted into the sites *Cla*I of pCV1 and pCV3 downstream EGFP and DsRed to produce the vectors pCV2 (P*_porB_*-EGFP) and pCV4 (P*_porB_*-DsRed), respectively. Plasmids pCV2 and pCV4 were separately transformed into the isolates LNP19995 and LNP21019. Fluorescence of transformants was confirmed using immunofluorescence microscopy and flow cytometry. One isolate of each transformation was selected and designated LNP19995-GFP (/Red) and 21019-GFP (/Red). These transformants were used in indicated experiments.

### Cell lines and infection

The human endometrial carcinoma Hec-1-B, the respiratory A549 and the laryngeal carcinoma HEp-2 epithelial cell lines and the mouse fibroblasts L929 cell lines were from American Type Culture Collection (Manassas, VA). The human cell lines (Hec-1-B, A549 and HEp-2 cells) and the L929 cells were maintained at 37°C under 5% CO_2_ humidified atmosphere in RPMI 1640 and DMEM, respectively, supplemented with 10% heat-inactivated fetal bovine serum (FBS). Cells were seeded in 24- or 96-well culture plates, at a density of 5×10^5^ or 5×10^4^ cells/well. Infection was carried out at bacteria to cell ratio of 10: 1 unless otherwise specified. All infection experiments and treatments were carried out in absence of serum to avoid the interference of the serum with PorB [17]. When indicated, cells were incubated with 1 µM STRP (used as positive control for apoptosis) or purified LOS equivalent to MOI 10. TNF-α was used at a final concentration of 5 ng/ml alone or in combination with 20 µg/ml cycloheximide. For antibody neutralizing experiments, cells were co-incubated for the indicated time with 100 µg/ml of the specific neutralizing antibody or isotype-matched irrelevant IgGs, or with 250 pg/ml of sTNF-RI (Monosan, France). Cells were then carefully washed several times with PBS before further analysis

### Adhesion assay and cytopathic effect determination

Adhesion assay was performed as described previously [66]. Cell viability was measured by Naphthol blue black (NBB) staining assay [67]. Cells infected in 96-well plates for the indicated time points as described previously, were stained with NBB solution (0.05% NBB in 9% acetic acid with 0.1 M sodium acetate) for 30 min at room temperature. The wells were washed three times with PBS to remove the free dye and fixed in 10% formalin for 5–10 min. The attached dye was resolved with 150 µl of 50 mM NaOH. The absorbance at 620 nm was measured by using a Multiskan Ascent Autoplate reader (Thermo Scientific, France). The percent of cytopathic effect was calculated as (1- A_620 nm_ of infected cells/A_620 nm_ of uninfected cells)×100.

### Analysis and quantitative measurement of apoptosis

Annexin V assay. Annexin V specifically binds to phosphatidylserine, a plasma membrane lipid that rapidly relocalizes from the inner leaflet to the outer leaflet in cells that are undergoing programmed cell death (apoptosis). Concomitantly, the extent of overall cytotopathogenicity was measured by the standard PI staining. Co-staining with Annexin V and PI allows differentiation of viable cells (Annexin V^−^, PI^−^) from early apoptotic cells (Annexin V^+^, PI^−^) and late apoptotic cells (Annexin V^+^, PI^+^). Cells infected in 24-well tissue culture plates as described earlier, were harvested using cold PBS/0.02% EDTA and washed twice in PBS. Double staining with FITC-Annexin V and Propidium iodide (PI) was carried out using the FITC-Annexin V kit, according to the manufacturer's recommendations and then analyzed by FACS. When indicated, apoptosis was also quantified using the ApoPercentage Apoptosis Kit according to the manufacturer's instructions.

Assay for mitochondrial permeability transition (MPT): To determine variations of mitochondrial permeability transitions (MPT), as indicative of apoptosis, cells were stained with the potential-sensitive dye 1,1′,3,3,3′,3′-hexamethylindodicarbo-cyanine iodide (DiIC1_(5)_) for flow cytometric analysis, as recommended by the manufacturer (Molecular Probes). This dye is reportedly only incorporated into mitochondria with intact membrane potential. After 20 min incubation with the dye at room temperature and darkness, cells were washed and analyzed by flow cytometry. Cells with high fluorescence intensity for DiIC1_(5)_ were considered as living cells. Cells with low fluorescence intensity for DiIC1_(5)_ were considered apoptotic cells.

Fluorometric analysis of caspase-3 activity. Following infection or STRP treatment, cells were harvested and processed for caspase-3 assay using caspase-3 assay kit, as recommended by the manufacturer. After incubation at 37°C for 70 min, fluorescence was measured at 405 nm by a microplate reader Multiskan Ascent, (Thermo Scientific, France). Standard p-Nitroaniline (pNA) solution was used for calculating caspase activity.

Extraction of cellular DNA and gel electrophoresis. Harvested cells were lysed for 1 h at 50°C in lysis buffer (0.1% Triton X-100, 5 mM Tris-HCl, pH 7.5; 0.5 mM EDTA, pH 7.2) supplemented with proteinase K (5 mg/ml) and digested for 1 h with RNase A (0.5 mg/ml). After two phenol/chloroform (1∶1) extractions, the DNA was precipitated with ethanol, dried, resolved in TE (10 mM Tris-Cl, pH 7.5, 1 mM EDTA pH 8), separated on a 1.5% agarose gel and visualized under ultra-violet light after ethidium bromide staining.

### Cell surface staining and flow cytometry

To measure cell surface expression of TNF-RI, cells were collected in HBSS containing 0.1% sodium azide (NaN_3_) and 1% FBS (staining buffer). Cells were labeled with anti TNF-RI mAb, anti-TNF-RII or irrelevant isotype-matched IgGs for 20 min. Cells were then washed twice with staining buffer and labeled with FITC-conjugated secondary antibody for 20 min. Cells were then fixed in 2% paraformaldehyde in staining buffer. Samples were analyzed using a FACSCalibur flow cytometer (BD Biosciences, France) equipped with FITC signal detector FL1-H (excitation = 488 nm, green). Relative fluorescence intensities were recorded from a total of 10,000 events. Results were analyzed using WinMDI 2.8 software.

### Fluorescence microscopy

The immunofluorescence staining was performed as described previously [68]. Briefly, cells adherent to glass coverslips were infected for the indicated time, then fixed in 2.5% paraformaldehyde in PBS and blocked with 1% normal goat serum in PBS. Anti-TNF-RI mAb was used at 10 µg/ml and rhodamine-conjugated goat anti-mouse IgG was used at a dilution of 1∶1000. Coverslips were mounted on microscope slides in ProLong Gold antifade reagent (Invitrogen) to minimize photobleaching. Slides were then examined by digital confocal microscopy using Zeiss Axio Imager. D1 fluorescent microscope coupled to AxioCam MRm vers.3 (Carl Zeiss, Germany). Digital images were acquired using appropriate filters and combined using the Axiovision Rel. 4.6 software (Carl Zeiss).

### Measurement of TNF-α secretion, TNF-α bioactivity, and TNF-α/TNF-RI complex

Supernatants of infected cells were collected, cleared of bacteria and cells by centrifugation at 14,000×*g* and kept frozen at −80°C until use. The amounts of TNF-α secreted in the supernatants were determined using the TNF-α enzyme-linked immunosorbent assay (ELISA) kit (R&D Systems, Abingdon, UK) in accordance with the manufacturer's instructions. TNF-α bioactivity was assessed by measuring cytopathic effect on L929 murine fibroblasts cells. In brief, L929 cell monolayers cultured for 24 h in 96-well plates (5×10^5^ cells/well), were overlaid with twofold serial dilutions of culture supernatants from Hec-1-B infected cells in RPMI 1640 supplemented with polymixin B to a final concentration of 1 µM to avoid the effect of free circulating LOS. After incubation at 37°C for 24 h the cells were carefully washed with PBS, and processed for NBB staining as described above. Estimates of the concentrations of bioactive TNF-α in the supernatants were obtained by comparison with calibration curves established with an rhTNF-α standard. TNF-α bioactivity in Hec-1-B supernatant samples was inhibited by anti–TNF-α Ab, but not by control rabbit IgG, indicating that the cytopathic activity in Hec-1-B supernatants represents TNF-α. TNF-α bound to TNF-RI was detected in supernatants of *N. meningitidis*-infected Hec-1-B cells by a mixed Ab ELISA. The supernatants were added to 96-well microtiter plates coated with an anti TNF-RI mAb, and, after 2 h at room temperature, the plates were washed and incubated for 1 h at room temperature with a rabbit anti-human TNF-α Ab (0.2 µg/ml). After washing, HRPO-conjugated goat anti rabbit IgG (Caltag) was determined. Peroxidase activity was determined by addition of citrate buffer and A_450_ was performed using a microplate reader. Calibrated dilutions of rhTNF-α captured by immobilized anti–TNF-α Ab were used as an internal standard for the comparative measurements of TNF-α complexed with TNF-RI.

### RNA isolation, RT–PCR, LOS, and PorB purification and Immunoblot

Total RNA was prepared using Total RNA isolation kit (Biolabs, France) according to manufacturer's recommendations. cDNA synthesis and PCR conditions were previously described (Deghmane et *al.*, 2002). To assess the expression analysis of *TNFRI* and *TNFRII* the primer pairs TNFR1-Fw/TNFR1-Rev, TNFR2-Fw/TNFR2-Rev were used (Table 2). β-actin-Fw/β-actin-Rev (Table 2) were used to amplify β-actin as internal control. Controls without RNA and/or reverse transcriptase were included in the assay. Meningococcal LOS was prepared with similar MOIs as previously described [69]. PorB was semi-purified as described by [16] and used in combination with 1 µM polymixin B to prevent any effect of LOS. For Immunoblot, cells were lysed 10 min on ice by adding RIPA lysis buffer [50 mm Tris-HCl (pH 7.4), 150 mm NaCl, 0.25% sodium deoxycholate, 1% NP40, 0.1% sodium dodecyl sulfate and freshly added protease Inhibitor Cocktail]. Whole cell lysates were then centrifuged at 13,000×*g* for 10 min at 4°C; and supernatants were collected to obtain protein extracts. Protein concentrations were determined by the Bradford assay (Bio-Rad, Hercules, CA). Fifty micrograms of extracted protein was run on SDS-PAGE and transferred to a nitrocellulose membrane. The blots were then probed overnight at 4°C with the relevant antibodies, washed and probed again with species-specific secondary antibodies coupled to HRPO and signal was detected by chemiluminescent reagents.

### Statistical analysis

Data are expressed as the mean±SD of triplicate samples, and the reproducibility was confirmed at least in three separate experiments. Statistical analysis were performed using student *t* test (two-way Annova) test, and considered significant if *P*<0.05.

## Supporting Information

Figure S1 DNA fragmentation in Hec-1-B cells following infection with pathogenic ST-11 isolates or carriage isolates of *N. meningitidis*. At 9 h after infection, epithelial cell DNA was extracted, separated in a 1.5% agarose gel, and stained with ethidium bromide. The 100-bp ladder is shown. Uninfected or STRP treated cells were used as controls.
(0.75 MB TIF)

Figure S2 LNP19995 but not LNP21019 induced apoptosis in A549 and Hep-2 epithelial cell lines. Cells were treated with TNF-α alone or in combination with CHX, or infected with either isolate. After 9 h of incubation in presence of anti-TNF-α neutralizing antibody (closed bars) or irrelevant antibody (Irr Ab, open bars), cells were stained with FITC-Annexin V and PI and apoptotic cells were analyzed by FACS. Data represent the mean±SD of three independent experiments. *** represent P<0.001 regarding untreated cells.
(0.16 MB TIF)

Figure S3 Level of TNF-RI increased in infected cells. (A) FACS analysis revealed up-regulation of intracellular level of TNF-RI at 9 h post-infection. The solid open histograms: unstained cells, the dotted histograms: isotype control stained cells, the gray-filled histograms anti-TNF-RI stained cells. (B) Consistently, immunoblot analysis showed a strong increase in the amount of TNF-RI protein, but not TNF-RII in infected or TNF-α-treated cells compared with uninfected cells.
(0.36 MB TIF)

Table S1 Summary of cytopathic and apoptotic features of meningococcal isolates used in this study.
(0.05 MB PDF)
