# Supplementary figures and images for: Differential Modulation of TNF-α–Induced Apoptosis by Neisseria meningitidis
Source: PLoS Pathog. 2009 May 1;5(5):e1000405. doi: 10.1371/journal.ppat.1000405 (PMC2669886; doi:10.1371/journal.ppat.1000405)

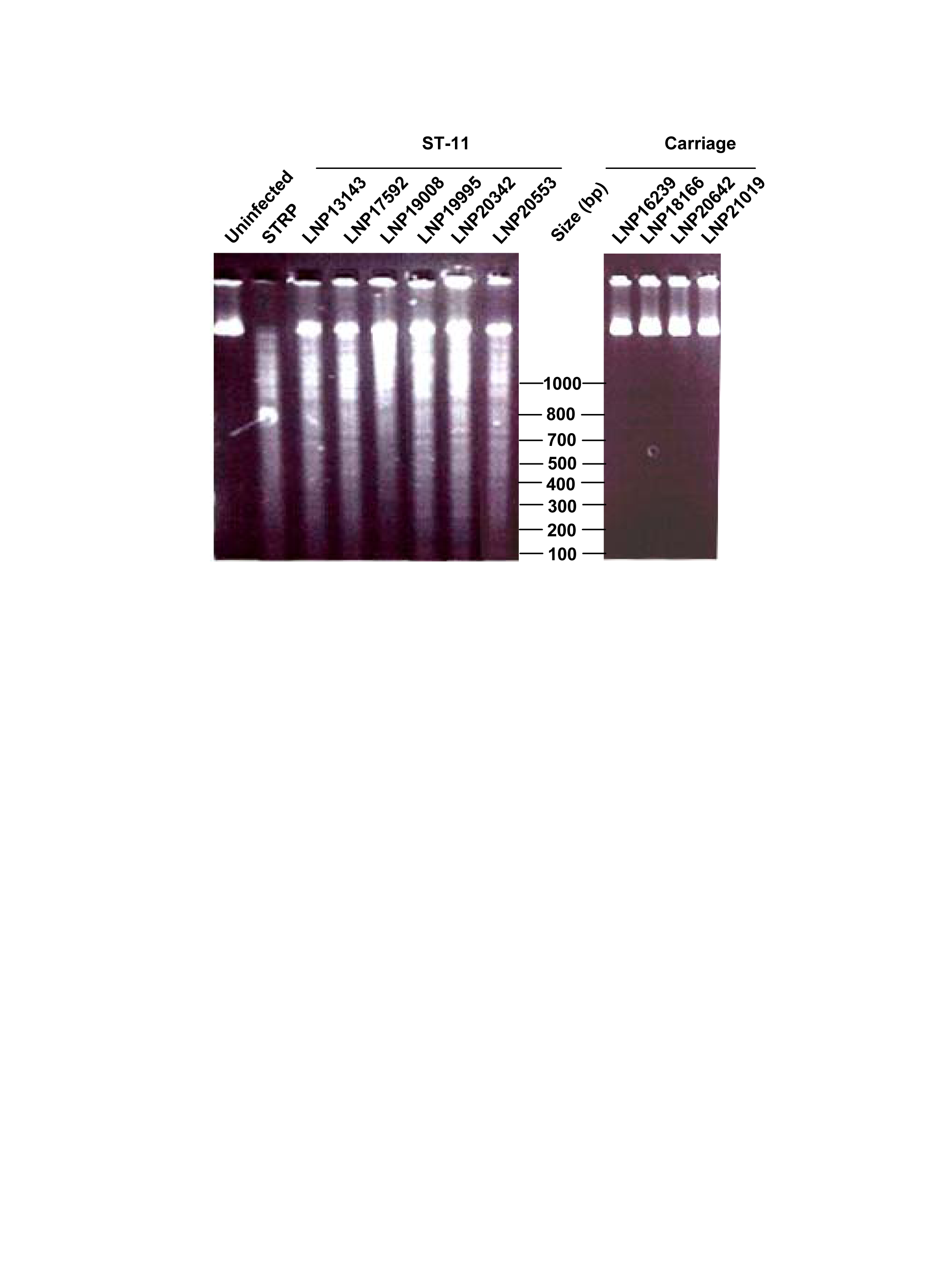

Supplement: Figure S1 — DNA fragmentation in Hec-1-B cells following infection with pathogenic ST-11 isolates or carriage isolates of N. meningitidis. At 9 h after infection, epithelial cell DNA was extracted, separated in a 1.5% agarose gel, and stained with ethidium bromide. The 100-bp ladder is shown. Uninfected or STRP treated cells were used as controls. (0.75 MB TIF) [file ppat.1000405.s001.tif]

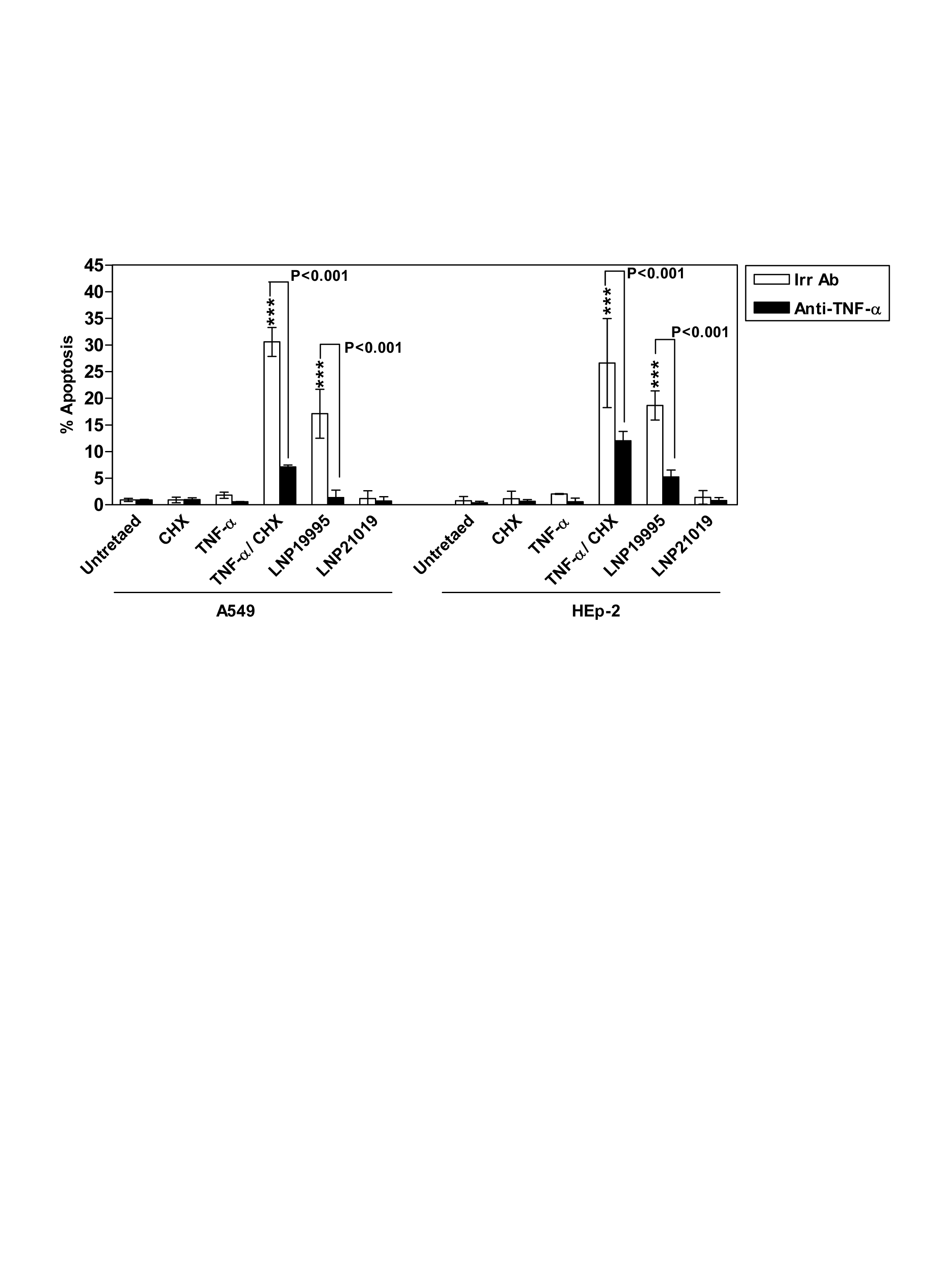

Supplement: Figure S2 — LNP19995 but not LNP21019 induced apoptosis in A549 and Hep-2 epithelial cell lines. Cells were treated with TNF-α alone or in combination with CHX, or infected with either isolate. After 9 h of incubation in presence of anti-TNF-α neutralizing antibody (closed bars) or irrelevant antibody (Irr Ab, open bars), cells were stained with FITC-Annexin V and PI and apoptotic cells were analyzed by FACS. Data represent the mean±SD of three independent experiments. *** represent P<0.001 regarding untreated cells. (0.16 MB TIF) [file ppat.1000405.s002.tif]

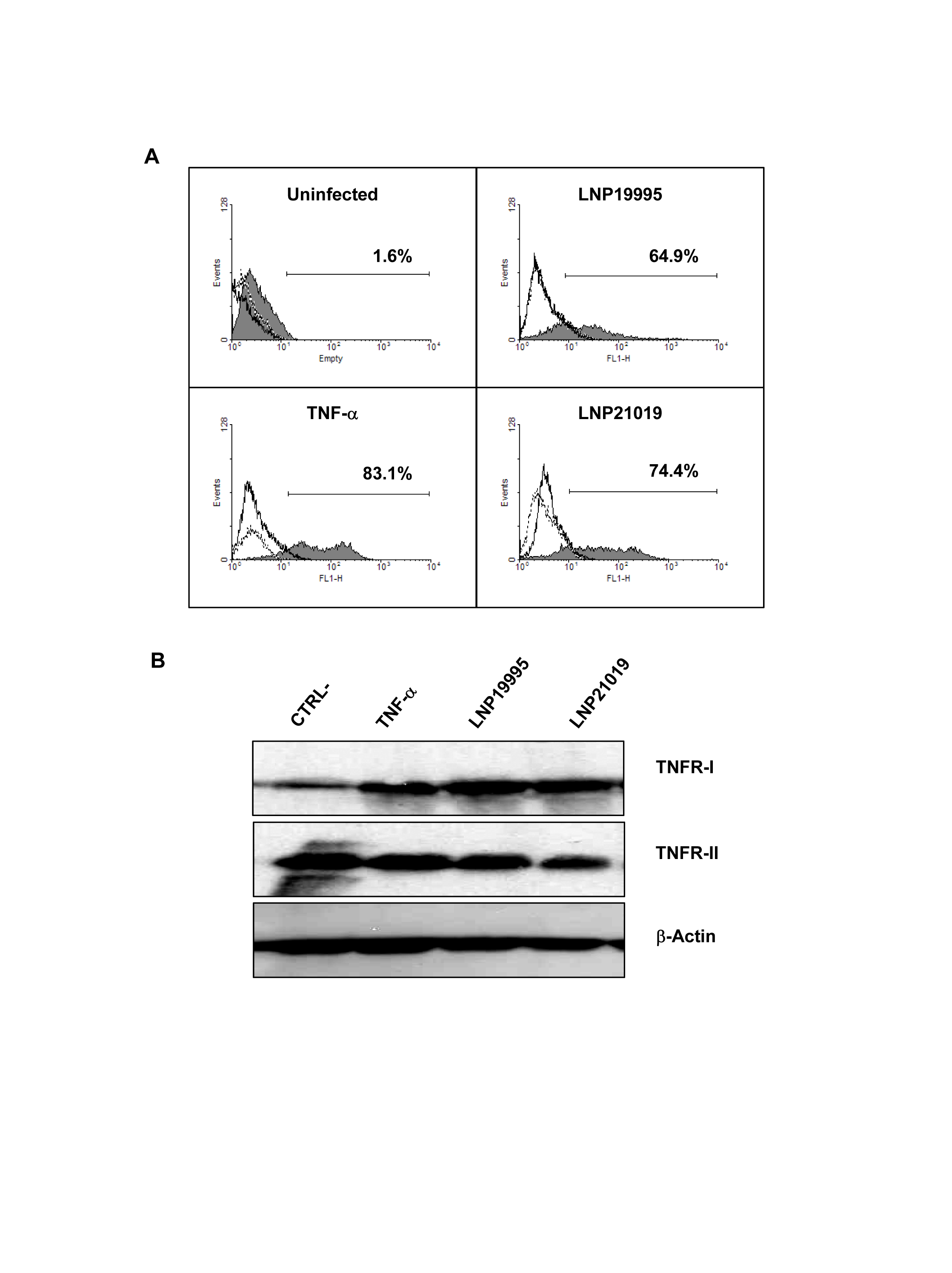

Supplement: Figure S3 — Level of TNF-RI increased in infected cells. (A) FACS analysis revealed up-regulation of intracellular level of TNF-RI at 9 h post-infection. The solid open histograms: unstained cells, the dotted histograms: isotype control stained cells, the gray-filled histograms anti-TNF-RI stained cells. (B) Consistently, immunoblot analysis showed a strong increase in the amount of TNF-RI protein, but not TNF-RII in infected or TNF-α-treated cells compared with uninfected cells. (0.36 MB TIF) [file ppat.1000405.s003.tif]
