# Supplementary material for: Differential Modulation of TNF-α–Induced Apoptosis by Neisseria meningitidis
Source: PLoS Pathog. 2009 May 1;5(5):e1000405. doi: 10.1371/journal.ppat.1000405 (PMC2669886; doi:10.1371/journal.ppat.1000405)
Supplement: Table S1 — Summary of cytopathic and apoptotic features of meningococcal isolates used in this study. (0.05 MB PDF) [file ppat.1000405.s004.pdf]

Table S1: Summary of cytopathic and apoptotic features of meningococcal isolates used in this study.

	Isolate	Clonal Complex	Cytotopathic effect (% Cell death)				Apoptosis	Caspase-3 Activity
			Time pi (h)			*P* value		
			1	9	24			
	Uninfected		3.5 ± 3.1	5.0 ± 2.3	8.3 ± 3.1	-	-	146.2 ± 19.2
	STRP		9.2 ± 7.9	60.1 ± 6.9	83.8 ± 1.1	< 0.001	+	1625.2 ± 284.5
**Invasive**	LNP13143	ST-11	10.5 ±3.5	47.7 ± 2.3	65.4 ± 0.6	< 0.001	+	630.2 ± 78.0
	LNP17592	ST-11	5.3 ±2.5	47.8 ± 5.9	62.6 ± 1.4	< 0.001	+	710.1 ±150.9
	LNP19008	ST-11	9.2 ±5.1	52.3 ± 1.3	62.4 ± 2.3	< 0.001	+	806.2 ± 30.2
	LNP19995	ST-11	9 ± 4.3	51.8 ± 0.9	63.5 ± 8.4	< 0.001	+	956.9 ± 9.7
	LNP20342	ST-11	12.3 ± 6.7	47.0 ± 9.4	57.9 ± 7.0	< 0.001	+	770.0 ± 184.9
	LNP20553	ST-11	11.7 ± 6.2	46.9 ± 6.5	55.8 ± 5.3	< 0.001	+	868.0 ±57.0
	LNP21515	ST-11	13.8 ± 8.3	32.3 ± 4.5	52.2 ± 4.0	< 0.001	+	773.2 ± 70.2
	LNP21678	ST-11	14.6 ± 5.9	47.5 ± 6.5	60.4 ± 8.8	< 0.001	+	841.6 ± 6.4
	LNP21996	ST-11	11 ± 3.5	51.2 ± 7.3	64.6 ± 7.6	< 0.001	+	775.1 ± 45.6
	LNP24198	ST-11	12.7 ± 6.5	47.4 ± 4.1	57.5 ± 7.3	< 0.001	+	806.2 ± 204.9
**Carriage**	LNP16239	NA	8.3 ± 1.3	9.2 ± 0.5	15.3 ± 1.0	> 0.05	-	100.5 ± 5.1
	LNP10820	ST-5	8.4 ± 4.1	12.5 ± 7.1	22.4 ± 2.5	> 0.05	-	98.6 ± 5.1
	LNP18166	ST-22	11.4 ± 6.2	10.2 ± 2.8	12.4 ± 5.2	> 0.05	-	100.5± 1.9
	LNP1288	ST-32	9.7 ± 1.7	8.8 ± 4.2	9.8 ± 5.9	> 0.05	-	99.8 ± 1.1
	LNP1934	ST-32	3.9 ± 4.2	7.6 ± 6.6	7.6 ± 3.0	> 0.05	-	97.1 ±2.7
	LNP3503	ST-32	6.9 ± 1.3	12.7 ± 4.4	11.2 ± 6.8	> 0.05	-	93.4 ± 2.2
	LNP21019	ST-35	10.6 ± 6.5	13.0 ± 4.7	13.8 ± 3.3	> 0.05	-	98.0 ± 3.0
	LNP20642	ST-334	6.9 ± 0.7	12.0 ± 2.2	13.2 ± 5.4	> 0.05	-	100.5 ± 5.1

P value: Student *t* test
